# The Causal Role of Ectopic Fat Deposition in the Pathogenesis of Metabolic Syndrome

**DOI:** 10.3390/ijms252413238

**Published:** 2024-12-10

**Authors:** Joseph A. M. J. L. Janssen

**Affiliations:** Department of Internal Medicine, Erasmus Medical Center (Erasmus MC), Dr. Molewaterplein 40, 3015 GD Rotterdam, The Netherlands; j.a.m.j.l.janssen@erasmusmc.nl; Tel.: +31-0612752413

**Keywords:** ectopic fat deposition, overnutrition, hyperinsulinemia, insulin resistance, metabolic syndrome, lipodystrophy, abdominal (visceral) obesity, type 2 diabetes

## Abstract

Consuming a “modern” Western diet and overnutrition may increase insulin secretion. Additionally, nutrition-mediated hyperinsulinemia is a major driver of ectopic fat deposition. The global prevalence of metabolic syndrome is high and growing. Within this context, people with congenital lipodystrophy often experience a severe form of metabolic syndrome. Evidence is increasingly supporting that subtle partial lipodystrophy plays an important role in the development of metabolic syndrome in the general population. In individuals in the general population with subtle partial lipodystrophy, as well as in those with congenital lipodystrophy, the subcutaneous adipose tissues are unable to accommodate surplus energy intake. In both conditions, (excess) fat is directed toward the liver, pancreas, and muscles, where it is deposited as ectopic fat, as this fat can no longer be stored in the “safe” subcutaneous fat depots. Ectopic fat depositions cause insulin resistance in the liver and muscles, as well as β-cell dysfunction in the pancreas. Support of a direct pathological role of ectopic fat deposition in this condition is further provided by the rapid normalization of hepatic insulin sensitivity and improvement in pancreatic β-cell function after marked reductions in ectopic fat depositions. Thus, ectopic fat deposition in the liver, pancreas, and muscles may play a causal role in the pathogenesis of metabolic syndrome even in the general population. As such, the prevention of ectopic fat deposition may reduce the risk of metabolic syndrome and mitigate its effects.

## 1. Introduction

In 1947, the French physician Jean Vague observed that people with obesity and diabetes or clinical signs of cardiovascular disease had a central body fat distribution (android obesity) [1]. In contrast, this was rarely associated with cardiovascular disease in people with gynoid fat accumulation [1]. Therefore, Vague postulated that excess fat, especially when stored on the trunk, is metabolically more damaging to the body than fat stored on the limbs [2]. This hypothesis has been supported by several cross-sectional and prospective studies [3]. Excessive visceral fat (even in lean adults) is not only to be associated with insulin resistance but also with metabolic risk factors for coronary disease [4]. Independent of obesity and family history of type 2 diabetes, a strong negative relationship exists between central abdominal fat (intra-abdominal plus abdominal subcutaneous fat) and whole-body insulin sensitivity, suggesting that central abdominal fat may be a strong marker of insulin resistance [5].

In 1988, Gerald Reaven suggested that the increased risk of coronary artery disease (CAD) in people with hypertension is due to the combination of hyperinsulinemia/insulin resistance, impaired glucose tolerance (IGT), increased plasma triglyceride concentration, and decreased HDL concentration, which are all risk factors for CAD [6]. Reaven hypothesized that these risk factors play a major role in the development of CAD in the general population [6]. Reaven further proposed that hyperinsulinemia/insulin resistance to insulin-stimulated glucose uptake plays a pivotal role in the pathogenesis and clinical course of three major related diseases: type 2 diabetes, hypertension, and CAD [6]. The co-occurrence of risk factors for both cardiovascular disease and type 2 diabetes mellitus suggests the existence of a syndrome, which was initially called Syndrome X and later replaced by the term “metabolic syndrome” [6]. Five unified criteria for diagnosing metabolic syndrome were published in 2009 (Table 1) [7]. When an individual meets at least three of the five proposed criteria, they qualify for a diagnosis of metabolic syndrome [7]. The prevalence of metabolic syndrome is high in adults and adolescents, and its incidence is increasing worldwide [8]. The global prevalence of metabolic syndrome varied from 12.5% to 31.4% between 1990 and 2018, depending on the applied diagnostic criteria [9]. From 1999 to 2014, the overall incidence of metabolic syndrome in the National Health and Nutrition Examination Survey (NHANES) increased from 27.6 to 32.3% [10]. Metabolic syndrome increases the relative risk of type 2 diabetes in the next 5 to 10 years approximately five-fold and the relative risk of cardiovascular disease approximately two-fold [11]. Many questions regarding the pathogenesis of metabolic syndrome remain unanswered despite extensive research [12].

Ectopic fat deposition is defined as the storage of excess triglycerides in tissues other than adipose tissue. For example, the liver, pancreas, skeletal muscles, and heart normally contain only small amounts of fat [13]. Ectopic fat deposition in the liver, pancreas, skeletal muscle, and heart can interfere with organ and tissue functions [13,14]. As such, ectopic fat deposition has been implicated in the pathogenesis of insulin resistance in the liver and muscles [15].

Evidence is increasingly suggesting that the combination of the consumption of a “modern” Western diet, overnutrition, early development of hyperinsulinemia, ectopic fat deposition, and insulin resistance plays an essential role in the pathogenesis of metabolic syndrome [16,17,18,19]. This review discusses how overnutrition, hyperinsulinemia, ectopic fat deposition, and insulin resistance play a pivotal role in the development of metabolic syndrome.

## 2. What Comes First: Insulin Resistance or Hyperinsulinemia?

**Figure 1:**
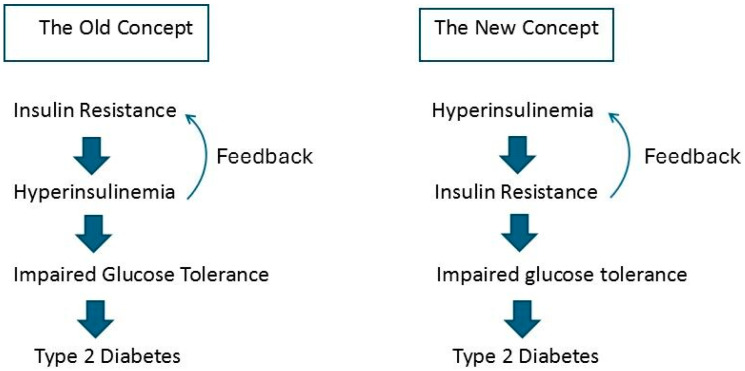
The old versus new concept of the role of hyperinsulinemia in the pathogenesis of insulin resistance. (**Left**): The old concept, which is still a widely held view, posits that insulin resistance is the primary cause of secondary hyperinsulinemia. Via feedback, hyperinsulinemia may further contribute to an increase in insulin resistance. This process may be followed over time by impaired glucose tolerance and, finally, frank type 2 diabetes due to pancreatic β-cell exhaustion. The old concept has been questioned, because it does not explain why hyperinsulinemia is present in people with normal glucose tolerance. (**Right**): According to the new concept, hyperinsulinemia due to (over) nutrition, genetics, and/or the environment is the primary cause of secondary insulin resistance when glucose tolerance is still normal. Through feedback, insulin resistance may further contribute to an increase in hyperinsulinemia. This process may be followed over time by impaired glucose tolerance and, finally, frank type 2 diabetes due to pancreatic β-cell exhaustion (see text for details).

The euglycemic hyperinsulinemic clamp is considered the gold standard for assessing insulin sensitivity in vivo [20]. During this clamp, plasma glucose concentrations are held constant at plasma insulin concentrations of approximately 100 μU/mL via infusing exogenous glucose [20]. The glucose infusion rate during the clamp is equal to the glucose uptake of all the tissues in the body under steady-state conditions during euglycemia [20]. The clamp therefore produces a measure of tissue sensitivity to (exogenous) insulin [20]. Insulin sensitivity widely varies (by more than six-fold) in apparently healthy nondiabetic populations [21]. People with low effective insulin-mediated glucose disposal during the euglycemic hyperinsulinemic clamp are considered insulin-resistant, meaning they are resistant to the glucose-lowering actions of insulin. This resistance to insulin-mediated glucose uptake occurs in >80% of individuals with impaired glucose tolerance (IGT) or type 2 diabetes mellitus and in at least 25% of individuals without obesity and with normal oral glucose tolerance [6]. Until recently, the prevailing view was that, in most cases, insulin resistance is the primary abnormality preceding hyperinsulinemia. Hyperinsulinemia was thought to be a compensatory response to insulin resistance; thus, in this old concept, insulin resistance was considered to be present before hyperinsulinemia clinically manifested [22,23] (Figure 1). Glucose levels remain within the normal range if increased insulin secretion is able to overcome insulin resistance [24]. In this old concept, insulin resistance was considered the initial defect leading to the development of metabolic syndrome, hyperglycemia, and type 2 diabetes [25]. However, this old concept has been recently questioned. In many prospective studies, fasting hyperinsulinemia in healthy normoglycemic individuals has been identified before the development of impaired glucose tolerance/type 2 diabetes at follow-up [26,27,28,29,30,31]. Moreover, insulin secretion was found to be elevated before the development of hyperglycemia in a longitudinal study of Rhesus monkeys with a form of type 2 diabetes, which is similar to that found in humans [32]. Understanding how insulin resistance is responsible for increased insulin secretion is difficult when blood glucose concentrations are normal [19]. The old concept did not satisfactorily explain why hyperinsulinemia causes obesity in insulin-resistant fat cells, because hyperinsulinemia is essential for the accumulation of lipids within insulin-sensitive tissues [33,34]. Moreover, only a few gene loci pointed to insulin resistance as the primary cause of type 2 diabetes in genome-wide association studies (GWASs), further decreasing the likelihood that insulin resistance is the primary abnormality preceding hyperinsulinemia [35]. Therefore, a new concept has been proposed: a chronically elevated insulin level (hyperinsulinemia) is the main driver of insulin resistance (Figure 1) [36,37,38]. Genetic predisposition, sustained excessive calorie intake (due to overnutrition), a Western dietary pattern, the consumption of food additives, and reduced hepatic insulin clearance may all contribute to fasting and postprandial hyperinsulinemia [19,36,39,40]. In support of this new concept, several prospective population-based studies conducted among people with normal glucose tolerance at baseline demonstrated that hyperinsulinemia precedes insulin resistance [36]. Further support of this new concept was published by Trico et al. [41]. They found that excessive insulin secretion in adolescents was associated with the development of obesity and glucose intolerance in later life, independent of insulin resistance [41]. Hyperinsulinemia in the absence of impaired glucose tolerance and normal HbA1c levels may be an early biomarker of the future risk of metabolic syndrome/type 2 diabetes [16,18].

Hyperinsulinemia-induced insulin resistance may act as a feedback control mechanism that limits the extent of hyperinsulinemia-mediated insulin receptor activation. In this scenario, insulin resistance thus functions as a physiological adaptation mechanism of the body that prevents hypoglycemia and maintains normoglycemia [19]. Hyperinsulinemia-induced insulin resistance may also function as a (physiological) defense mechanism protecting critical tissues against fuel overload, insulin-mediated metabolic stress, and overnutrition-induced injury (see below) [42,43].

In 2019, Richard Bergman proposed an alternative hypothesis regarding the cause of hyperinsulinemia and insulin resistance. Bergman postulated that (inherited) reduced hepatic insulin clearance causes hyperinsulinemia in the peripheral circulation [44]. Bergman further suggested that peripheral (i.e., posthepatic) hyperinsulinemia, via the downregulation of insulin receptors, induces insulin resistance in the skeletal muscles. In the long term, this may result in pancreatic β-cell failure and the development of frank diabetes in people who are susceptible [44]. Thus, according to Bergman’s hypothesis, reduced hepatic insulin clearance is the primary event that leads to the subsequent development of (peripheral) hyperinsulinemia and insulin resistance. In support of this hypothesis, Tamura found impaired insulin clearance and hyperinsulinemia in apparently healthy Asian people without obesity or notable insulin resistance, suggesting that this change may be an initial trigger that drives subsequent insulin resistance [45].

## 3. The Subcutaneous Tissue Functions as a (Temporary) Reservoir of Excess Energy Under Healthy Conditions

The white adipocytes of the subcutaneous tissue are normally highly plastic, being able to switch between two opposing metabolic programs: nutrient storage and nutrient release [46,47]. The ability of adipocytes to store calories as triglycerides when food intake is in excess of the immediate energy needs is a biological adaptation that has provided a strong evolutionary advantage [48]. The adipocytes in the subcutaneous (white) fat depots are initially insulin-sensitive and involved in the regulation of plasma glucose levels [49]. In lean people, insulin stimulates glucose uptake and modulates adipocyte lipid metabolism via stimulating free fatty acids uptake and de novo fatty acid synthesis, as well as inhibiting lipolysis [49,50]. In the healthy state, the subcutaneous fat depots function as reservoirs or “metabolic sinks” that store excess energy in the form of triglycerides [51]. The triglycerides stored in the adipocytes of the subcutaneous fat depots can be converted in the postprandial period into free fatty acids and glycerol, providing energy on demand [52]. Thus, excess calories can be “safely” stored in subcutaneous adipocytes as triglycerides after a short-term (acute) energy overload [53].

## 4. Subcutaneous Tissue and Its Capacity to Expand During Chronic Overnutrition

**Figure 2:**
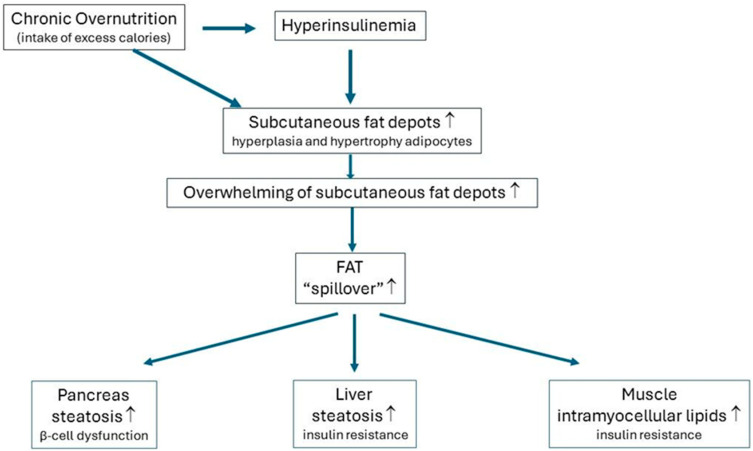
Simple scheme showing how chronic overnutrition and hyperinsulinemia may lead to fat spillover and ectopic fat depositions in the liver, pancreas, and skeletal muscles. Excess calorie intake chronically exceeding energy expenditure stimulates hyperinsulinemia. Excess calories are initially stored in the subcutaneous fat depots due to overnutrition-mediated hyperinsulinemia. Consequently, the subcutaneous fat depots start to expand to accommodate excess calories: the adipocytes of the subcutaneous fat depots develop hyperplasia and/or hypertrophy. However, the capacity of the subcutaneous adipocytes to accommodate the surplus energy intake is limited. Eventually, the individual-specific maximal storage capacity of the subcutaneous fat depots is reached. Fat spillover occurs from this moment on: Fat is directed from the subcutaneous fat depots toward other organs and then is ectopically deposited in the liver, pancreas, and skeletal muscles. Consequently, the liver and skeletal muscles may become insulin-resistant over time, whereas the pancreatic β cells may lose their ability to produce sufficient insulin after a glucose load. This mechanism may explain how the combination of chronic overnutrition and hyperinsulinemia is linked to the development of insulin resistance and pancreas dysfunction (see the text for more details). ↑ levels increase.

Under chronic excess calorie intake and hyperinsulinemia in an individual, the subcutaneous fat stores start to expand via (1) the differentiation and proliferation of pluripotent stem cell precursors (preadipocytes) into small mature adipocytes (hyperplasia) and/or (2) increasing the size (hypertrophy) of the mature adipocytes. Hyperplasia increases the number of fat-storing adipocytes [54,55]. The capacity to expand subcutaneous fat stores (by hyperplasia and/or hypertrophy) markedly varies between individuals, is partly genetically determined, and is sex-specific (less capacity in men) [47,54,56,57,58]. The maximal capacity to store excess calories in the subcutaneous fat stores was hypothesized to be individual-specific [54]. The subcutaneous fat depots no longer function as “safe” metabolic sinks when the maximal subcutaneous adipose tissue expansion capacity of an individual is reached [47,54]. A high-caloric intake/modern Western diet where calorie intake chronically exceeds the rate of caloric expenditure causes hyperinsulinemia (Figure 2). Overnutrition-mediated hyperinsulinemia initially stimulates the storage of excess calories in the subcutaneous fat depots. Consequently, the subcutaneous fat stores start to expand to accommodate the supply of excess calories: the adipocytes of the subcutaneous fat depots develop hyperplasia and/or hypertrophy. However, subcutaneous adipocytes have a limited capacity to accommodate excess calories. Eventually, the maximal storage capacity of the subcutaneous fat depots is reached. After this, the spillover of extra energy occurs in the form of lipids (i.e., free fatty acids), and fat is directed from the subcutaneous fat depots toward other organs [15]. As such, a chronically high calorie intake leads to ectopic fat deposition; abnormal amounts of triglycerides are stored in the visceral adipose tissue, liver, pancreas, and skeletal muscles (Figure 2) [59]. A calorie intake that chronically exceeds energy expenditure, in combination with low physical activity, is the main cause of ectopic fat deposition in the liver, pancreas, and skeletal muscle in the Western world [15]. Moreover, ectopic fat may be deposited in individuals both with and without obesity [60,61].

As a direct result of ectopic fat deposition, (1) the liver and skeletal muscles may develop insulin resistance over time, and (2) pancreatic β-cells may become dysfunctional, which may collectively lead to the metabolic abnormalities that are characteristic of metabolic syndrome and type 2 diabetes (Figure 2; see below) [45,62,63,64].

## 5. Patients with Congenital Lipodystrophy Typically Present with a Severe Form of Metabolic Syndrome

Lipodystrophy is a syndrome characterized by severe insulin resistance, the complete or partial absence of subcutaneous (white) adipose tissue, a lack of visceral fat, and pathological fat deposition (ectopic fat) at a distinct anatomical site [65,66,67,68,69]. People with a severe form of congenital lipodystrophy typically have reduced subcutaneous gluteofemoral and leg fat, ectopic fat depositions in the liver and other tissues, severe insulin resistance, hypertriglyceridemia, and type 2 diabetes [70]. In addition, hypertension and cardiovascular disorders, including atherosclerosis, are highly prevalent in people with lipodystrophy [71]. Thus, patients with congenital lipodystrophy often experience a severe form of metabolic syndrome [72]. The severity of insulin resistance in patients with congenital lipodystrophy is broadly proportional to the extent of subcutaneous fat loss [72]. The insulin resistance, nonalcoholic fatty liver disease, hypertriglyceridemia, and type 2 diabetes found in people with congenital lipodystrophy are thought to be directly related to ectopic fat deposition in the liver, pancreas, and skeletal muscles [65,66,72,73]. The results obtained from several animal models of congenital lipodystrophy support the concept that the loss of subcutaneous fat and ectopic fat deposition may cause fat infiltration into the liver, pancreas, and skeletal muscles, along with insulin resistance, glucose intolerance, and diabetes [74].

AZIP/F1 lipodystrophic mice lack virtually all white adipose tissue and develop a severe form of insulin-resistant diabetes that is comparable to that in humans with congenital generalized lipodystrophy [75]. The adipose tissue of normal wild-type mice was transplanted into AZIP/F1 lipodystrophic mice to test whether the metabolic abnormalities seen in humans and mice with generalized lipodystrophy are a direct consequence of the loss of adipose tissue mass [76]. The transplantation of the wild-type adipose tissue reversed hyperglycemia, considerably reduced (i.e., normalized) plasma insulin levels and hepatic steatosis, and increased muscle insulin sensitivity [76]. Moreover, the benefits of adipose tissue transplants were dose-dependent and required near-normal amounts of transplanted fat [76]. The results of this experiment demonstrated that the insulin resistance and diabetes in this model are caused by ectopic fat deposition and a lack of subcutaneous adipose tissue [76]. In another experiment, white adipocyte progenitor cells (preadipocytes) were transplanted into AZIP/F1 lipodystrophic mice; these cells reconstituted the normal white adipose tissue depot mass, which rescued the diabetic phenotype of these animals [77]. Thus, these data collectively provide evidence that a lack of both subcutaneous adipose tissue and ectopic fat deposition plays an important role in the pathogenesis of insulin resistance and type 2 diabetes.

## 6. Reduced Subcutaneous Adiposity and Ectopic Fat Deposition May Be a Central Mechanism in the Development of Insulin Resistance and Metabolic Syndrome

The prevalence of metabolic syndrome is high in children and adolescents with obesity [66]. Children and adolescents with obesity often show a phenotype reminiscent of partial lipodystrophy. In addition, they present with hepatic steatosis, a high proportion of visceral fat, a relatively low proportion of abdominal subcutaneous fat, severe insulin resistance, and an increased risk of metabolic syndrome [66,78]. Many of the characteristics of people with congenital lipodystrophy can also be found in individuals diagnosed with metabolic syndrome. Several population-based studies have provided evidence that subtle partial lipodystrophy, which is characterized by reduced subcutaneous adiposity, may contribute to the pathogenesis of insulin resistance and metabolic syndrome in the general population [58,72,79,80,81,82]. In the U.K. Biobank imaging and Fenland prospective cohort studies, which were prospective population-based studies involving detailed metabolic phenotyping, the genome-wide polygenic score for fat mass ratio was associated with an increased risk of metabolic syndrome and cardiometabolic diseases [83]. The fat mass ratio, defined as dual X-ray absorptiometry (DEXA)-based trunk fat percentage (trunk fat %) divided by leg fat percentage (leg fat %), is a method of discriminating partial lipodystrophy in the general population [83]. A lipodystrophy-like phenotype was present in approximately one in eight participants in the UK Biobank cohort and one in twenty participants in the Fenland study cohort [83]. However, a comprehensive clinical assessment is necessary to diagnose a lipodystrophy-like phenotype, where DEXA-based measures can be supportive but not definitive on their own [84]. Scott et al. used fasting insulin levels as a proxy for insulin resistance, finding that more than half the genetic loci associated with higher fasting insulin levels were also associated with reduced gluteofemoral fat mass as measured via DEXA scanning, higher triglyceride levels, and lower HDL cholesterol levels [80]. In another population-based study, Lotta et al. found that 53 common genetic variants associated with higher fasting insulin levels were associated with lower levels of gynoid and leg fat mass, higher triglyceride levels, lower HDL cholesterol levels, and type 2 diabetes [81]. Moreover, individuals who carried these 53 common genetic variants showed an impaired capacity to increase hip fat mass and were more likely to develop insulin resistance and type 2 diabetes during chronic overnutrition [81]. Collectively, these data suggest that partial subtle lipodystrophy may be a major factor in the pathogenesis of insulin resistance/metabolic syndrome in the general population during chronic overnutrition.

Lotta et al. demonstrated that the same 53 common genetic variants contributed to familial partial lipodystrophy-type 1, which is a condition characterized by an extreme form of severe insulin resistance [81]. Thus, common genetic variants show considerable overlap between individuals with common and mild insulin resistance in the general population and people with congenital lipodystrophy [81,82]. The inability of subcutaneous adipose tissue depots to accommodate surplus energy intake and ectopic fat deposition seems to be the common denominator between both conditions [58,79,80,81,82]. However, most of these studies discussed were observational and therefore could not answer whether the metabolic disturbances in individuals with common/mild insulin resistance in the general population are secondary to adipose repartitioning or are a result of a direct effect of genetic variants. In addition, genetic factors are not the sole drivers of the relationship between ectopic fat deposition and metabolic syndrome development. Other factors, such as age, sex, ethnicity, physical activity, diet composition beyond caloric intake, and hormonal factors such as cortisol levels, are potential confounders and may influence the relationship between ectopic fat deposition and metabolic syndrome development.

The existence of subtle partial lipodystrophy in individuals within the general population fits well within the personal fat threshold hypothesis, which postulates that each individual has a personal fat threshold that determines their susceptibility to developing impaired glucose tolerance/type 2 diabetes [85]. The personal fat threshold is crossed when an individual of any body mass index can no longer store triglycerides in the metabolically safe subcutaneous fat depots [85]. This promotes excess liver fat accumulation and hepatic fat export, as well as exposes pancreatic β cells to excess lipids. Taylor et al. postulated that these are the main etiological factors in the development of impaired glucose tolerance/type 2 diabetes [85]. The personal fat threshold hypothesis was confirmed in the ReTUNE study [86]. This study showed that cycles of 5% rapid weight loss corrected excess intrahepatic fat levels, reduced hepatic fat export, induced the recovery of β-cell function, increased glycemic control, and led to a return to a nondiabetic metabolic state in the majority of individuals with a BMI < 27 kg/m^2^ [86]. Further support of a causal role of ectopic fat deposition in the pathogenesis of insulin resistance/metabolic syndrome has been provided by studies demonstrating that marked reductions in ectopic fat deposition can reverse insulin resistance in the liver and muscles [87]. The recovery of normal hepatic insulin sensitivity was observed in people with type 2 diabetes after considerable weight loss and substantial reductions in intrahepatic fat deposition [88].

## 7. Further Evidence of the Role of Ectopic Fat Deposition in the Development of Insulin Resistance/Metabolic Syndrome

Fabbrini et al. found that hepatic insulin resistance was primarily related to intrahepatic lipid content and not to visceral fat mass, although the amount of visceral adipose tissue correlated with the intrahepatic lipid content [89,90]. The molecular mechanism by which ectopic fat causes hepatic insulin resistance continues to be debated [91]. The putative mechanism through which ectopic fat depositions cause hepatic insulin resistance is not via the accumulation of free fatty acids in the liver but rather via the accumulation of one of its metabolites, diacylglycerol (DAG) [92,93]. This is consistent with the findings of animal and human studies, where the liver DAG content was approximately five-fold higher in individuals with hepatic insulin resistance and hepatic steatosis than in individuals without hepatic insulin resistance [93]. The accumulation of DAG in the liver promotes the translocation and activation of protein kinase C-ε (PKC-ε), which, in turn, inhibits insulin receptor kinase activity [92,93]. As such, DAG inhibits insulin-stimulated insulin receptor substrate (IRS)-1 tyrosine phosphorylation, which results in the reduced activity of IRS-1-associated phosphatidyl inositol 3 kinase (PI3K) of the insulin receptor [88]. This may ultimately interfere with the insulin-induced activation of glycogen synthesis and the suppression of glucose production in the liver [15]. The hypothesis that intracellular DAG directly contributes to insulin resistance in the liver is further supported by findings in people with and mouse models of lipodystrophy [94]. Teleologically, hepatic insulin resistance induced by DAG represents a mechanism for stopping further energy storage in the liver when hepatic intracellular lipids are already in excess [15]. Rats fed a high-fat diet for 3 days accumulated hepatic fat and developed hepatic insulin resistance prior to the development of peripheral tissue insulin resistance, suggesting that hepatic insulin resistance is the primary event that leads to the subsequent development of peripheral tissue insulin resistance [95]. Samuel et al. reported that the short-term high-fat feeding of rats provides a suitable model for studying the effects of hepatic fat accumulation on hepatic insulin responsiveness that excludes the potentially confounding effects of peripheral tissue insulin resistance [96]. This short-term high-fat feeding of rats caused an approximately three-fold increase in liver triglyceride and total fatty acyl-CoA contents without any notable increase in the visceral or skeletal muscle fat content [96]. In addition, the suppression of hepatic glucose production by insulin was diminished in the rats after short-term feeding of a high-fat diet, despite normal insulin-stimulated peripheral glucose disposal [96]. Samuel et al. demonstrated a dose–effect relationship between hepatic fat accumulation and insulin resistance [96]. Hepatic insulin resistance could be attributed to impaired insulin-stimulated IRS-1 and IRS-2 tyrosine phosphorylation, as these changes were associated with the activation of PKCε [96]. Moreover, hepatic fat accumulation resulted in the decreased insulin activation of glycogen synthase and increased gluconeogenesis, similar to that found in people with impaired glucose tolerance/type 2 diabetes [96]. The development of fatty liver and hepatic insulin resistance, the activation of PKC-ε, and defects in insulin receptor signaling were all abrogated when rats fed a high-fat diet were treated with the mitochondrial uncoupler 2,4-dinitrophenol to increase energy expenditure [96]. This result suggests that the hepatic insulin resistance in these rats was specifically dependent on hepatic fat accumulation [96]. Overall, these data support the hypothesis that ectopic fat deposition leads to hepatic insulin resistance. The findings of Samuel et al. for rats agreed with Taylor’s twin cycle hypothesis [15,97]. Taylor postulated that long-term overnutrition (i.e., a positive calorie balance) in humans increases insulin secretion and stimulates ectopic fat deposition in the liver [97]. In Taylor’s hypothesis, ectopic fat deposition in the liver induces hepatic insulin resistance, decreases hepatic insulin clearance, and increases hepatic glucose production, thereby increasing the basal peripheral (posthepatic) plasma insulin levels [97]. Hyperinsulinemia stimulating de novo lipogenesis in the liver produces a self-reinforcing cycle (twin cycle hypothesis) [97,98,99]. De novo lipogenesis in the liver normally has three possible outcomes: (1) lipids can be oxidized as energy, (2) lipids can be stored in the liver, or (3) lipids can exported as VLDL-TG in the plasma to be delivered to other body tissues [98]. VLDL-TG export by the liver is the main outcome of de novo lipogenesis when the liver is saturated with fat due to ectopic fat deposition [100]. The VLDL-TG exported by the liver contributes to ectopic fat deposition in other tissues of the body, including the pancreas and skeletal muscles [100]. Long-term exposure to saturated fatty acids may be harmful to normal pancreatic β-cell function, especially in individuals with a specific genetic susceptibility to fat-induced β-cell dysfunction. This may contribute to the development of impaired glucose tolerance/type 2 diabetes [101]. In animals, ectopic fat manifests in the skeletal muscles in the form of lipid droplets [102]. Intramyocellular lipid (IMCL) in the skeletal muscles impairs insulin receptor substrate-1 and phosphatidylinositol 3-kinase, which activates glucose transport and glycogen synthesis [94]. IMCL in human skeletal muscles is associated with decreased insulin sensitivity independent of the overall obesity and fat distribution [103]. Understanding which molecular mechanisms link IMCL to changes in insulin signaling is vital for the development of new therapies for treating insulin resistance. Shulman et al. hypothesized that an increase in the intramuscular content of DAG in the skeletal muscles stimulates the activation of protein kinase C-theta (PKC-ϴ) [104]. The increase in the intramuscular content of DAG is a result of an imbalance between fatty acid delivery and intracellular fatty oxidation and storage in the skeletal muscles [104]. Increases in the intramuscular DAG content stimulate the activation of protein kinase C-theta (PKC-ϴ) in the skeletal muscles [104]. The activation of PKC-ϴ, in turn, decreases insulin-stimulated insulin receptor substrate-1/insulin receptor substrate-2 (IRS-1/IRS-2), tyrosine phosphorylation, phosphatidinylinosostol-3 kinase (PI3K) activation, and downstream insulin signaling [104]. This results in reduced glycogen synthesis in the muscle owing to the reduced insulin-mediated translocation of glucose transporter-4 (GLUT-4) to the plasma membrane [104]. Collectively, the findings of the studies discussed in this section support an important role of ectopic lipid deposition/metabolites in the pathogenesis of insulin resistance/metabolic syndrome in humans.

## 8. Preventing the Accumulation of and Losing Ectopic Fat

The main goal of metabolic syndrome treatment should be to prevent or to alleviate the energetic overload of subcutaneous adipose tissue depots. Therefore, reducing the daily caloric intake should be a mainstay of the treatment of metabolic syndrome. Eating a healthy, low-calorie diet, exercising regularly, and maintaining a normal weight are appropriate and proven strategies for preventing ectopic fat deposition. The consumption of a Mediterranean diet is protective against ectopic fat accumulation and reduces the prevalence of metabolic syndrome and the incidence of cardiovascular disease [105,106,107,108,109,110,111]. A meta-analysis found that lifestyle interventions can reduce ectopic fat deposition in the internal organs of people with overweight and obesity [112]. A considerable decrease in ectopic fat deposition of approximately 5% was observed in the liver and pancreas after increasing physical activity or starting a hypocaloric diet, and a trend toward decreased intramyocellular lipid levels was observed [112]. Two weeks of exercise training decreased the pancreatic fat content and strengthened pancreatic β-cell function, regardless of baseline glucose tolerance [113]. In another study, moderate weight loss achieved with diet alone or in combination with exercise did not substantially reduce the muscle lipid stores despite a significant increase in insulin sensitivity, suggesting that muscle ectopic lipid depositions are less sensitive to lifestyle interventions than the liver and pancreas [114]. In addition, individuals with relatively large depositions of ectopic fat have a low chance of benefiting from lifestyle interventions alone [112]. More intensive strategies may be required for these individuals. In individuals with type 2 diabetes, body weight losses of over 10–15 kg were needed to normalize the amount of fat within the liver and pancreas. This reversed hepatic insulin resistance, increased pancreatic insulin secretion, and lowered fasting and postprandial hyperglycemia [115]. Moreover, severe weight reductions (~15–24% of the body weight at baseline) achieved with an 800 kcal diet or after bariatric surgery reduced the muscle lipid content in individuals with obesity and morbid obesity [116,117]. Bariatric surgery resulted in comparable long-term weight loss and improvements in metabolic comorbidities in individuals with or without metabolic syndrome [118]. The prevalence of metabolic syndrome decreased after bariatric surgery from 66.2% to 3.5%, whereas the Homeostatic Model Assessment for Insulin Resistance score decreased from 77.5% to 22.5% at follow-up [119]. However, not all people with metabolic syndrome are eligible for bariatric surgery, despite its efficacy in treating certain patient subgroups [120]. In addition, the use of bariatric surgery in the general population as a first-line treatment is not currently viable because of the cost and the additional requirements for personnel and equipment [120,121]. No pharmacological treatment has yet been approved by the FDA or EMEA that specifically targets ectopic fat and completely reverses metabolic syndrome. Liraglutide, the glucagon-like peptide-1 (GLP-1) receptor agonist, reduces appetite and caloric intake. The daily administration of liraglutide for 36 weeks to adults with overweight or obesity with metabolic syndrome, as an adjunct to a reduced-calorie diet and increased physical activity, resulted in substantially lower visceral and ectopic fat levels compared to those in the placebo group [122]. In another study, daily liraglutide administration decreased intrapancreatic fat deposition in people with type 2 diabetes with a fatty pancreas [123]. Tirzepatide is a glucose-dependent insulinotropic polypeptide (GIP) and GLP-1 receptor agonist, which is administered weekly. Tirzepatide also reduces appetite and caloric intake. In the SURPASS-3 study, tirzepatide induced notable reductions in the liver, visceral, and subcutaneous fat levels, as assessed with magnetic resonance imaging [124]. In the SURMOUNT-1 study, three years of treatment with tirzepatide in people with obesity and prediabetes resulted in substantial and sustained weight reduction [125]. In addition, tirzepatide treatment resulted in a 94% reduction in the risk of progression to type 2 diabetes compared to the placebo from baseline to week 176 of treatment [125]. However, the currently available data on the effects of GLP-1 analogs on ectopic fat/metabolic syndrome are limited. More evidence and information are needed before these drugs can be considered as potential options for the treatment of ectopic fat deposition and metabolic syndrome. In addition, more clarity is required regarding the long-term risk/benefit balance and cost-effectiveness of treatment with these drugs.

A healthy lifestyle combined with a low-calorie diet, weight loss for those with overweight or obesity, and regular physical exercise are currently the only proven nonpharmacological therapies that reduce ectopic fat deposition and its associated risks. However, the effects of these lifestyle interventions are moderate.

The worldwide increase in the prevalence of metabolic syndrome underscores the urgent need to develop new, affordable, low-cost, and effective strategies both at the population level and for individuals to prevent and treat metabolic syndrome and mitigate its associated risks. Simple and cheap methods must be developed to identify and regularly monitor individuals with excess ectopic fat deposition in clinical practice. A recent study demonstrated the utility of serum lipidomics as a biomarker for ectopic fat deposition: serum lipidomics were particularly powerful in predicting the presence of intramuscular metabolites of free fatty acids (DAG and ceramide) in insulin-resistant humans [126]. In another study, the results of untargeted metabolomics revealed several plasma metabolites that were predictive of ectopic fat depositions in the pancreas and liver, as assessed with magnetic resonance imaging [127]. The prevention of and reduction in hyperinsulinemia should be scrutinized to evaluate the related reductions in the risk of metabolic syndrome and the lessening of its effects, because hyperinsulinemia is an independent risk factor for ectopic fat deposition/metabolic syndrome.

Attempts should be aimed at developing new treatments that are able to increase the fat storage capacity of the peripheral (subcutaneous) adipose tissue depots and/or to reduce selectively ectopic fat depositions in the liver, pancreas, and muscles. Whether these newly developed treatments can also improve long-term outcomes should be further tested.

## 9. Concluding Remarks

**Figure 3:**
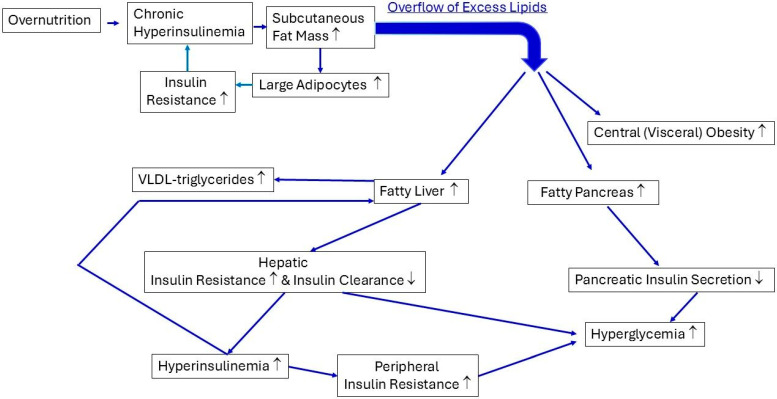
Scheme showing the involvement of chronic overnutrition and hyperinsulinemia in the development of ectopic fat depositions in the liver, pancreas, and abdomen, as well as the influence of ectopic fat depositions on the function of these organs. Overnutrition-mediated chronic hyperinsulinemia stimulates the expansion of the subcutaneous fat mass. This is accompanied by enlargement in the adipocytes (hypertrophy). Large (hypertrophied) adipocytes may develop insulin resistance, which may intensify the lipolysis of the fat cells and contribute to the further worsening of hyperinsulinemia. When the maximal expansion capacity of the subcutaneous adipose tissue has been reached over time, due to the chronic positive energy balance, the subcutaneous adipose tissue can no longer function as a “safe” metabolic sink. This causes an overflow of excess lipids, which accumulate as ectopic fat depositions in the abdomen, liver, and pancreas. The ectopic fat depositions in the abdomen manifest as central (visceral) obesity. The ectopic fat depositions in the liver cause hepatic insulin resistance and decrease hepatic insulin clearance. Individuals with subtle partial lipodystrophy are more prone to developing ectopic fat depositions. Hepatic insulin resistance and decreased hepatic insulin clearance both contribute to hyperinsulinemia and thus to the development of peripheral (posthepatic) tissue insulin resistance in the skeletal muscles. Hepatic insulin resistance causes higher fasting glucose levels because of the decreased insulin-mediated suppression of hepatic glucose production. In addition, the combination of chronic hyperinsulinemia and fatty liver leads to the increased production and export of very-low-density lipid-triglycerides (VLDL-TGs) by the liver. The VLDL-TGs exported by the liver contribute to ectopic fat depositions in the pancreas and skeletal muscles (not shown in Figure 2). Ectopic fat depositions in the pancreas are harmful for pancreatic β-cell function, and this may lead to the long-term loss of insulin secretion, hyperglycemia, and frank diabetes (see the text for more details). Note: ↑, levels increase; ↓, levels decrease.

The prevalence of metabolic syndrome continues to increase worldwide. Hyperinsulinemia occurs because of genetic predisposition, sustained overnutrition, the consumption of a Western diet, and reduced hepatic insulin clearance. Hyperinsulinemia is an early biomarker that predicts a future risk of metabolic syndrome. The combination of overnutrition and hyperinsulinemia stimulates the storage of excess calories in the “safe” subcutaneous fat depots. Figure 3 summarizes how overnutrition and hyperinsulinemia are involved in the ectopic fat deposition in the liver, pancreas, and abdomen. Overnutrition-mediated chronic hyperinsulinemia initially promotes the expansion of the subcutaneous fat depots. This expansion of the subcutaneous fat depots is associated with adipocyte hypertrophy. Hypertrophied adipocytes may become insulin-resistant, which intensifies the lipolysis of the fat cells, which contributes to a further increase in hyperinsulinemia/insulin resistance. Excess lipids “overflow” when the maximal expansion capacity of the subcutaneous adipose tissue is reached due to the chronic positive energy balance. This leads to ectopic fat depositions in the abdomen, liver, and pancreas. These ectopic fat depositions manifest in the abdomen as central (visceral) obesity. Ectopic fat depositions in the liver cause hepatic insulin resistance and decrease hepatic insulin clearance. The hepatic insulin resistance and decreased hepatic insulin clearance both contribute to hyperinsulinemia and thus to the development of peripheral tissue insulin resistance in the skeletal muscles. Hepatic insulin resistance induces higher fasting glucose levels due to the decreased insulin-mediated suppression of hepatic glucose production. In addition, the combination of chronic hyperinsulinemia and fatty liver leads to the increased production and export of very-low-density lipid-triglycerides (VLDL-TGs) by the liver. The VLDL-TGs exported by the liver and transported in the circulation contribute to ectopic fat deposition in the pancreas and skeletal muscles. Ectopic fat deposition in the pancreas is harmful to pancreatic β-cell function; this may lead, in the long term, to the loss of insulin secretion, hyperglycemia, and frank diabetes.

Marked reductions in ectopic fat deposition may result in the reversal of insulin resistance in the liver and muscles and normalization of pancreatic β-cell function. Thus, ectopic fat deposition may play a causal role in the pathogenesis of metabolic syndrome. The prevention of ectopic fat deposition may be essential in reducing the risk of metabolic syndrome and mitigating its consequences.

## Figures and Tables

**Table 1:** The criteria for the diagnosis of metabolic syndrome *.

Increased waist circumference, with ethnic-specific waist circumference cut-off points
Fasting plasma glucose ≥ 100 mg/dL (5.6 mmol/L) or previously diagnosed type 2 diabetes
Systolic blood pressure ≥ 130 mm Hg, diastolic blood pressure ≥ 85 mm Hg, or treatment for hypertension
High-density lipoprotein (HDL) cholesterol < 40 mg/dL (1.03 mmol/L) in men or <50 mg/dL (1.29 mmol/L) in women or treatment for low HDL levels
Triglyceride levels ≥ 150 mg/dL (1.7 mmol/L) or treatment for elevated triglyceride levels

* Modified from Alberti et al. [7].

## Data Availability

Not applicable.
